# Silica Bottle Resonator Sensor for Refractive Index and Temperature Measurements

**DOI:** 10.3390/s16010087

**Published:** 2016-01-09

**Authors:** Galina Nemova, Raman Kashyap

**Affiliations:** 1 Department of Engineering Physics, Polytechnique Montréal, P.O. Box 6079, Station Centre-ville, Montréal, QC H3T 1J4, Canada; raman.kashyap@polymtl.ca; 2 Department of Electrical Engineering, Polytechnique Montréal, P.O. Box 6079, Station Centre-ville, Montréal, QC H3T 1J4, Canada

**Keywords:** refractive index sensor, temperature sensor, bottle resonator

## Abstract

We propose and theoretically demonstrate a bottle resonator sensor with a nanoscale altitude and with alength several of hundreds of microns made on the top of the fiber with a radius of tens microns for refractive index and temperature sensor applications. The whispering gallery modes (WGMs) in the resonators can be excited with a taper fiber placed on the top of the resonator. These sensors can be considered as an alternative to fiber Bragg grating (FBG) sensors.The sensitivity of TM-polarized modes is higher than the sensitivity of the TE-polarized modes, but these values are comparable and both polarizations are suitable for sensor applications. The sensitivity ~150 (nm/RIU) can be reached with abottle resonator on the fiber with the radius 10 μm. It can be improved with theuse of a fiber with a smaller radius. The temperature sensitivity is found to be ~10 pm/K. The temperature sensitivity can decrease ~10% for a fiber with a radius *r_co_* = 10 μm instead of a fiber with a radius *r_co_* = 100 μm. These sensors have sensitivities comparable to FBG sensors. A bottle resonator sensor with a nanoscale altitude made on the top of the fiber can be easily integrated in any fiber scheme.

## 1. Introduction

**Figure 1:**
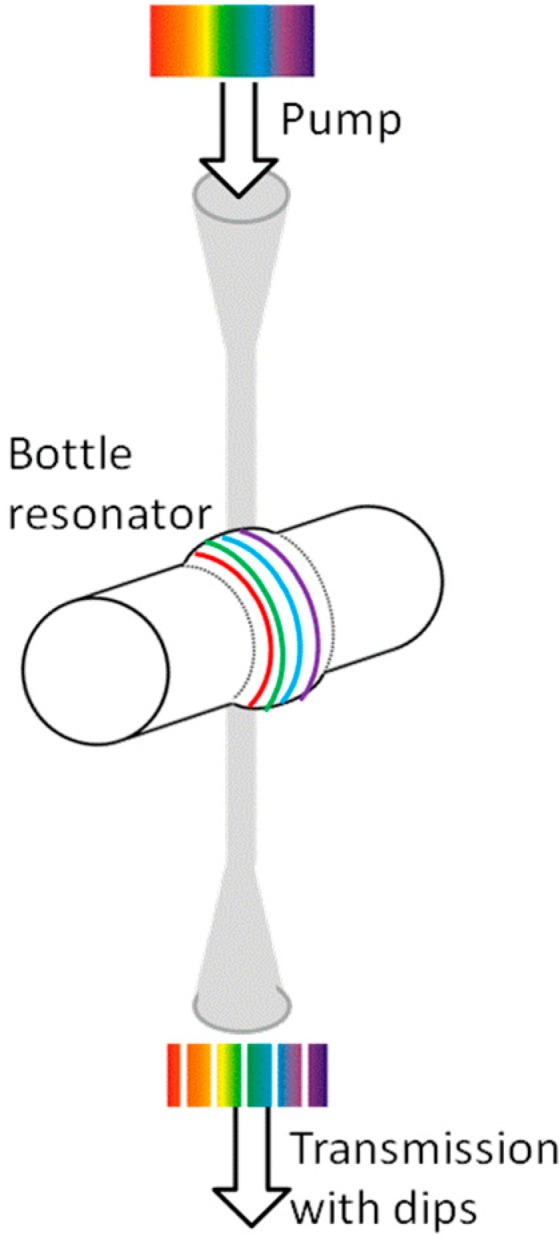
Structure under investigation: a fiber with bottle resonator is excited with a tapered fiber. The dips in the output spectrum correspond to the WGMs circulating in the resonator.

A bottle resonator made on the surface of the optical fiber is a smooth parabolic perturbation of the fiber radius with a nanoscale altitude, which looks like a bottle. Operation of the bottle resonator is based on whispering gallery modes (WGMs) circulating on the surface of the resonator perpendicular to the fiber axis.The parabolic thickness profile of the bottle resonator, like a linear harmonic oscillator, provides light confinement along the fiber axis (Figure 1). Similarly to the electromagnetic field of surface plasmon-polaritons (SPPs) the electromagnetic field of WGMs is localized near the surface of the resonator. This field distribution makes WGMs useful for sensor applications [1,2,3]. Contrary to SPP devices [4,5,6,7,8], WGM devices are completely dielectric, that is free from metal components which exhibit loss such as in metal films or particles.

In this paper we consider a silica fiber bottle resonator with a nanoscale altitude for refractive index and temperature sensing applications. WGMs of a bottle resonator can be excited with the evanescent field of biconically tapered fiber (Figure 1). The excited WGMs appear as transmission dips in the output spectrum of a tapered fiber. The shift of these dips with the change in the refractive index or temperature can be used for sensing applications. In order to position our sensors amongst others let us consider the sensitivity of several widely used sensors, for example, fiber Bragg grating (FBG), WGM, and surface plasmon resonance (SPR) sensors. The temperature resolution of a FBG sensor is closely connected with the thermo-optic coefficient of the fiber. For example, for silica with its small thermo-optical and thermal expansion coefficients, the temperature sensitivity is ~10 pm/K at 1550 nm [9]. The refractive index sensitivity of FBG sensors depend on the fiber diameter, which increases for a smaller fiber diameter. For example, for a fiber with diameter of 2 μm the refractive index sensitivity is ~231.4 nm/RIU [10]. The refractive index sensitivity of SPR sensors is significantly higher. As an example for prism-coupled and grating-coupled SPR sensors it is ~7000 nm/RIU and ~3000 nm/RIU, respectively [11]. In [12] it has been shown theoretically that the temperature sensitivity of SPR sensors as high as 4 nm/K can be achieved. A comprehensive review of the current state of the art of physical and biological WGM sensors can be found in Ref. [13].In this review paperit has been shown that as in the case of FBG sensors a choice of the resonator material of WGM sensors is a crucial factor in their design. As an example of recent WGM sensor achievements it is worth mentioning the crystalline MgF_2_ disc resonator with a sensitivity of 1.09 nm/RIU. Refractive index sensitivities of 30, 570, and 700 nm/RIU have been reported in a microsphere resonator, a capillary-based optofluidic ring resonator, and a nanowire loop resonator, respectively [13]. Typically, in today’s WGM resonators the detection limit is 1.2 × 10^−6^ RIU [13]. A temperature sensitivity of 0.212 nm/K for WGMs in a fiber-based loop cavity has been reported [13]. The thermal response of Nd^3+^-doped barium titano-silicate glass microspheres has also been recently explored, and a tuning of 10 pm/K was demonstrated [13]. In [14], WGM temperature sensors with an associated detectable resonance wavelength shift of 1.56 × 10^−4^ pm around 1531 nm wavelength and with an approximate WGM temperature sensitivity of 14 pm/K at near room temperatures have been presented. It has been shown, theoretically, that the minimum resolvable temperature can be as small as 1.11 × 10^−5^ K [14]. A theoretical description of the bottle resonator sensor operation is presented in Section 2. The results of the simulations are discussed in Section 3.

## 2. Theoretical Analysis

In this part of the paper we give a short overview of the theory used to simulate the operation of proposed sensors. A bottle resonator can be described with a truncated harmonic-oscillator profile [15]:

(1) $$R\left(z\right)=R_{b}{\left[1+{\left(\Delta kz\right)}^{2}\right]}^{-1/2}$$

where *R_b_ = r_co_ +* Δ*r_co_*, *r_co_* is the radius of the fiber without of a resonator, Δ*r_co_* is the maximum altitude of the resonator. Δ*k* is a parameter, which can be obtained, for example, from an experiment. The electric field of a bottle resonator mode in the scalar approximation in adiabatical approximation in cylindrical coordinates (r,φ,z) can be presented as [16]:

(2) $$E\left(r,\varphi ,z\right)=\Psi_{m,p,q}\left(z\right)\Phi_{m,p}\left(r,z\right)\exp\left(im\varphi \right)$$

where an integer *m* (*m* = 0,1,2,…) is an azimutal number. It gives the number of field nodes around the circumference. An integer *p* (*p* = 1,2,…) is a radial quantum number. It gives the number of power maxima along the radius, and *q* (*q* = 0,1,2,…)is the discrete or continuous axial quantum number. Here:

(3) $$\Phi_{m,p}\left(r,z\right)=Ai\left[\frac{2^{1/3}m^{2/3}}{r_{co}}\left(r_{co}-r\right)-\alpha_{p}\right]$$

where *α_p_* is *p-*th root of the Airy function [17]. The amplitude *Ψ_m,p,q_*(z) in the case of a harmonic oscillator profile can be estimated using the one-dimensional Schrӧdinger equation [15,16,18] and described by the relation:

(4) Ψm,p,q(z)=[ΔEmπ22q+1(q!)2]14Hq(ΔEm2z)exp(−ΔEm4z2)

where *H_q_*(x) is the Hermite polynomial. Δ*E_m_* = 2*U_m,p_*Δ*k*/*R_b_*. *U_m,p_* can be estimated with the relation [19,20]:

(5) $$U_{m,p}\approx m\left[1+\frac{\alpha_{p}}{2^{1/3}m^{2/3}}-\frac{n_{cl}}{m{\left(n_{co}^{2}-n_{cl}^{2}\right)}^{1/2}}{\left(\frac{n_{co}}{n_{cl}}\right)}^{\pm 1}+\frac{3}{10}\cdot \frac{\alpha_{p}^{2}}{2^{2/3}m^{4/3}}\right]$$

Signs + and – correspond to TE and TM polarization, respectively. *c* is the speed of light in vacuum. *n_co_* and *n_cl_* are refractive index of the fiber and surrounding medium, respectively. In the first approximation $r_{co}k_{r}n_{co}\approx m$, where *k_r_* = *ω_r_*/c = 2π/*λ_r_*, and the WGM frequency, *ω_r_*, can be estimated using the geometry of a sample. This frequency corresponds to the condition for constructive interference of the wave upon a round trip of the resonator. The resonant wavelength of the WGM is

(6) $$\lambda_{m,p,q}=2\pi n_{co}{\left[{\left(\frac{U_{m,p}}{R_{b}}\right)}^{2}+\left(q+\frac{1}{2}\right)\Delta E_{m}\right]}^{-1/2}$$

In the case of the bottle resonator a smooth (nm) parabolic perturbation of the fiber radius can be described as

(7) $$R\left(z\right)=r_{co}+\Delta r\left(z\right)=r_{co}+\Delta r_{co}-\frac{z^{2}}{2R},\mathrm{for}\;0<z<L$$

where *L =* (*2R∆r_co_*)^1/2^ is the length of resonator. *R* is the radius of the curvature of the bottle resonator. As one can see in Equations (1) and (7) ${\left(\Delta k\right)}^{2}=\frac{2\Delta r_{co}}{R_{b}L^{2}}$. Following [13] the WGM excitation process can be simulated with the *δ*-function *C*δ(*z-z_c_*), where *C* is the coupling parameter. *z_c_* is the point near the top of the resonator on the *z*-axis, which is directed along the fiber axis, where the tapered fiber touches the resonator. In this case [18],

(8) $$\Psi_{m,p,q}\left(z\right)=CG\left(\lambda ,z_{c},z\right)$$

and the bottle resonator Green’s function can be presented as

(9) $$G\left(\lambda ,z,z\right)=\frac{\cos\left[\psi (\lambda ,z_{t1},z_{c})+\pi /4\right]\cos\left[\psi (\lambda ,z_{c},z_{t2})+\pi /4\right]}{2\beta \left(\lambda ,z_{c}\right)\cos\left[\psi (\lambda ,z_{t1},z_{t2}\right]}$$

where

(10) $$\psi (\lambda ,z_{c},z)=\int_{z_{c}}^{z}\beta \left(\lambda ,z\right)dz$$

Here, β(λ,z) is the propagation constant and the *z_t1_* and *z_t2_* are turning points, where *β*(*λ,z_t_*_1,2_) = 0 [18]. The WGM does not propagate beyond these points along the length of the fiber. We want to emphasize that the semiclassical theory fails near the turning points, since the axial wavelength, which is proportional to *β*^−1^(*λ,z_t_*_1,2_), reaches infinity at the turning points [19].

## 3. Results and Discussion

### 3.1. WGMs of the Bottle Resonator

**Figure 2:**
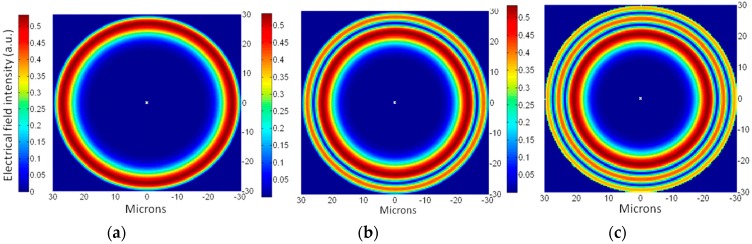
The electric field intensity distribution along the fiber radius for (**a**) *p* = 1, $\lambda_{m,1,0}$ = 1.4526 µm, (**b**) *p* = 2, $\lambda_{m,2,0}$ = 1.3948 µm, and (**c**) *p* = 3, $\lambda_{m,3,0}$ = 1.3597 µm. *r_co_* = 30µm, *m* = 176.

Let us consider a silica fiber with the radius *r_co_* = 30 μm. Following Equation(2) one can simulate the field distribution along the radius of the fiber for different modes (Figure 2). All calculations have been performed in Matlab with double precision. As one can see in Figure 2, the maximum of the field moves closer to the fiber axis as the radial quantum number *p* increases, that is, the WGM with *p* = 1 is the most suitable mode for sensing applications.

**Figure 3:**
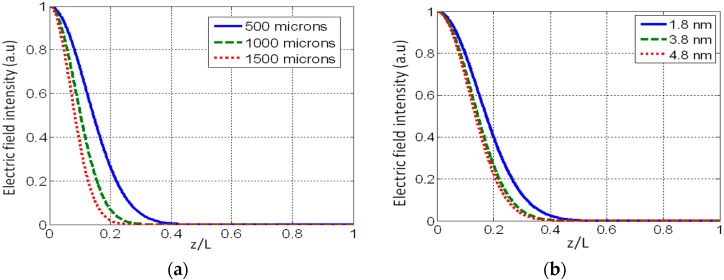
(**a**) The electric field intensity distribution along the length of the resonators with ∆*r_co_* = 3.8 nm and *L* = 500, 1000, and 1500 μm; (**b**) the electric field intensity distribution along the length of the resonators with ∆*r_c_*_o_ = 1.8, 2.8, and 3.8 nm and *L* = 500 μm. *n_cl_ =*1.33.

As we already mentioned, the bottle resonator is like a linear harmonic oscillator provides light confinement along the fiber axis. Using relation Equation (4) we have simulated the electric field intensity distribution in WGM along the length of the resonator (*z*-axis) with ∆*r_co_* = 3.8 nm, *n_cl_ =* 1.33. The resonators with three different lengths *L* = 500, 1000, and 1500 μm have been considered (Figure 3a). We have also simulated the electrical field intensity distribution in the WGM along the length of the resonator with *L* = 500 μm and three different altitudes ∆*r_co_* = 1.8, 3.8, and 4.8 nm (Figure 3b). As one can see in Equation (4) the WGM field becomes more concentrated near the top of the resonator with increasing ∆*r_co_*/*r_co_* and/or with decreasing length of the resonator, *L*. As an example, if ∆*r_co_* = 3.8 nm and the length *L* = 500 μm the WGM field is concentrated in the vicinity of 0.4 of the length of the resonator that is ~200 μm near the top of the resonators (Figure 3a). If the length of the resonator is increased up to *L* = 1500 μm and the altitude is the same ∆*r_co_* = 3.8 nm the WGM field is concentrated in the vicinity 0.23 of the length of the resonator that is ~345 μm near the top of the resonators (Figure 3a). If the altitude of the resonator is increased keeping a constant length *L* = 500 μm, the field of the WGM will be concentrated closer to the top of the resonator. For example if ∆*r_co_* = 1.8 nm the field is concentrated in the vicinity of 0.5 of the length of the resonator that is ~250 μm near the top of the resonator. For ∆*r_co_* = 4.8 nm this distance decreases to 0.4 of the length of the resonator that is ~200 μm near the top of the resonator.

### 3.2. Refractive Index Sensing

As one can see in relations Equations (5) and (6) the wavelengths, $\lambda_{r}=\lambda_{m,p,q}$ (renamed here for simplicity), of the WGMs are functions of the refractive index of the surrounding medium. WGMs circulate on the surface of the resonator. They have to be sensitive to any changes in the refractive index of the surrounding medium like SPPs. Each excited WGM appears as a transmission dip in the output spectrum of the tapered fiber (Figure 1). This dip will shift along the wavelength axis as the refractive index of the surrounding medium changes. This shift, ∆λ, divided by the corresponding change in the refractive index, ∆n, characterizes the sensor’s sensitivity ∆λ/∆n. The sensitivity of a bottle resonator sensor is different for TE and TM modes. It can be estimated from relations Equations (5) and (6) as

(11) $${\frac{d\lambda }{dn_{cl}}|}_{TE}\approx \frac{\lambda^{3}}{4\pi^{2}R_{b}}\left[\frac{U_{m,p}}{R_{b}}+\Delta k\left(q+\frac{1}{2}\right)\right]\frac{n_{cl}}{n_{co}{\left(n_{co}^{2}-n_{cl}^{2}\right)}^{3/2}}$$

for TE modesEquation (11), and

(12) $${\frac{d\lambda }{dn_{cl}}|}_{TM}\approx \frac{\lambda^{3}}{4\pi^{2}R_{b}}\left[\frac{U_{m,p}}{R_{b}}+\Delta k\left(q+\frac{1}{2}\right)\right]\frac{n_{cl}\left(2n_{co}^{2}-n_{cl}^{2}\right)}{n_{co}^{3}{\left(n_{co}^{2}-n_{cl}^{2}\right)}^{3/2}}$$

for TM modes Equation (12).

For our proposed structures Δ*k*<<*U_m,p_*/*R_b_* Equations (11) and (12) can be simplified as

(13) $${\frac{d\lambda }{dn_{cl}}|}_{TE}\approx \frac{\lambda^{2}}{2\pi r_{co}}\frac{n_{cl}}{{\left(n_{co}^{2}-n_{cl}^{2}\right)}^{3/2}}$$

for TE modes, and

(14) $${\frac{d\lambda }{dn_{cl}}|}_{TM}\approx \frac{\lambda^{2}}{2\pi r_{co}}\frac{n_{cl}\left(2n_{co}^{2}-n_{cl}^{2}\right)}{n_{co}^{2}{\left(n_{co}^{2}-n_{cl}^{2}\right)}^{3/2}}$$

For TM modes, respectively. 

**Figure 4:**
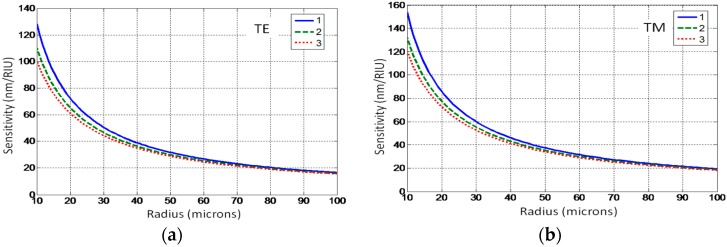
The sensitivity of the bottle resonator sensor as a function of the fiber radius for (**a**) the TE and (**b**) the TM polarized WGMs. *p* = 1, 2, and 3.

Figure 4 illustrates the sensitivity of the bottle resonator to the refractive index as a function of the fiber radius for TE and TM-polarizations. In our simulations the length *L* = 500 μm and the altitude ∆*r_co_* = 3.8 nm, and coupling constant |C|^2^ = 2 × 10^4^ m^−1^ [18]. The radius of the curvature of the resonator is *R* ≈ 32.8 m. As one can see in Equations (11) and (12) the sensitivity of the WGMs with TM-polarization is better than the sensitivity of the TE-polarized WGMs, although these values are comparable (Figure 4). The sensitivity of the first mode with *p* = 1 is better than the sensitivity of the second *p* = 2 and third *p* = 3 modes. Indeed, as we already mentioned the maximum of the WGM with *p* = 1 is the nearest to the surface (Figure 2a). Although the sensitivities of the WGMs with *p* = 2 and *p* = 3 are high enough to be useful for sensing applications. As one can see in Equations (13) and (14), the sensitivity of all modes decreases with increasing fiber radius (Figure 4). The sensitivities of all modes become almost equal to each other for fibers with *r_co_* > 60 μmradius. The decrease in the sensor’s sensitivity with the increase in the fiber radius is caused by the change in the field distribution along the fiber radius as the fiber radius increases. Indeed, for the fiber with the radius *r_co_* = 10 μm the maximum of the WGM intensity is located within ~0.71 μm of the fiber surface. For a fiber with the radius *r_co_* = 100 μm the maximum of the WGM intensity is located ~1.7 μm away from the fiber surface. This shift of the maximum of the field decreases the sensor’s sensitivity. As one can see from simulations based on Equations (13) and (14) the refractive index sensitivity changes in the range ~150–20 (nm/RIU) for TM modes and ~130–18 (nm/RIU) for TE modes for fibers, which have a radius belonging to the range 10–100 μm, respectively. That is, fibers with smaller radii are more favourable for the increase of the sensor sensitivity. It is easy to estimate that for a sensor with a refractive index sensitivity of~150 nm/RIU and an OSA’s resolution of 10 pm, the detection limit for refractive index is ~6.67 × 10^−5^.

### 3.3. Temperature Sensing

**Figure 5:**
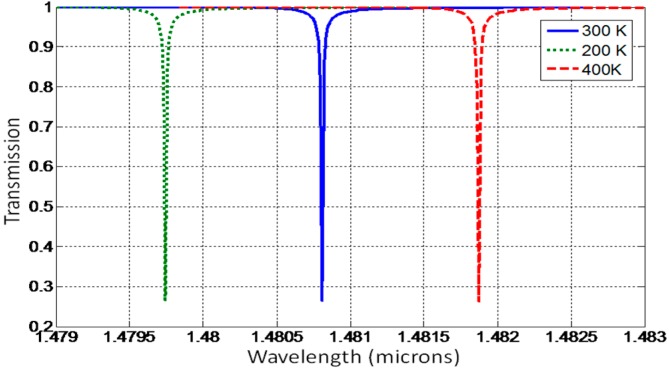
The transmission spectrum of the tapered fiber as a function of the wavelength for the temperatures 300, 200, and 400 K. *r_co_* = 30 μm, *L* = 500 μm, and ∆*r_0_* = 3.8 nm.

**Figure 6:**
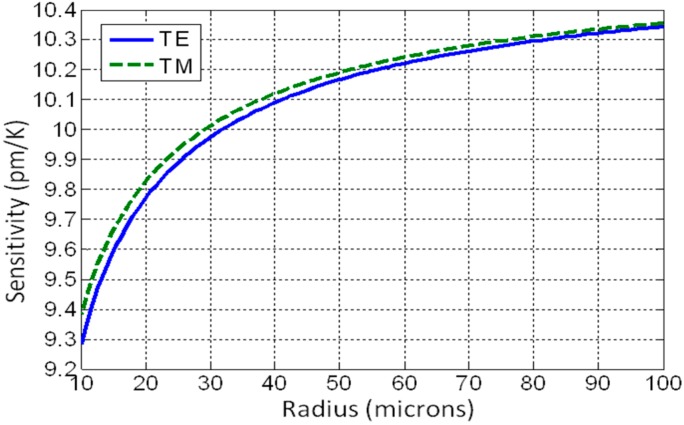
The sensitivity of the bottle resonator temperature sensor as a function of the fiber radius for TE and TM polarizations. *r_co_* = 30 μm, *L* = 500 μm, and ∆*r_0_* = 3.8 nm.

The WGM wavelength is a function of the refractive index and the radius of the fiber (see Equations (5) and (6)), which are functions of temperature, *i. e.*,a bottle resonator sensor can be used as a temperature sensor. Let us investigate its sensitivity to temperature. We assume that the sensor is placed in air or vacuum that is *n_cl_* = 1. The shift in the resonant wavelength with the temperature can be estimated in the first approximation as

(15) $$\Delta \lambda =\lambda_{r}\left(\alpha +\frac{1}{n}\frac{dn}{dT}\right)\Delta T$$

where ∆*T* is the change in the temperature. α=dr/(rdT) is the coefficient of thermal expansion, which is the fractional increase in radius per unit rise in temperature. It changes slightly with temperature in the range between ~0.2 × 10^−6^ K^−1^ at −50 °C and ~0.7 × 10^−6^ K^−1^ at 250 °C [21]. dn/dT is the thermo-optical coefficient. The thermo-optic coefficient of silica at room temperature is *dn*/*dT* ≈ 9.2 × 10^−6^ K^−1^. It decreases more or less linearly down to ~3 × 10^−6^ K^−1^ at liquid nitrogen temperature [22]. This dependence of the thermo-optical coefficient on the temperature has been taken into account in our simulations. As one can see in Equation (15) the influence of thermal expansion on the sensor sensitivity is less than the influence of the thermo-optic effect by a factor of approximately ten. As we see from our simulations the influence of the thermal expansion on the sensor’s sensitivity, which can be described as the relation:

(16) *S_T_* = ∆*λ*/∆*T*

is negligible in comparison with the thermo-optic effect and can be neglected in simulations. As before let us consider the bottle resonator sensor with the length *L* = 500 μm and the altitude ∆*r_0_* = 3.8 nm, and the coupling constant |C|^2^ = 2 × 10^4^ m^−1^. The transmission spectra of the tapered fiber for three different temperatures of the bottle resonator 200 K, 300 K, and 400 K have been simulated using the Green’s function Equation (9). They are presented in Figure 5. As one can see in Figure 5 the dip shifts with temperature. The bandwidths of the dips in the transmission spectrum are ~0.025 nm. The sensitivity of the bottle resonator as a temperature sensor can be estimated with Equations (15) and (16). The temperature sensitivity of the sensor as a function of the fiber radius is illustrated in Figure 6 for TM and TE polarized modes. The temperature sensitivity decreases ~10% as the fiber radius decreases from *r_co_* = 100 μm to *r_co_* = 10 μm. The decrease in the sensor sensitivity is caused by the decrease in the resonant wavelength, *λ_r_*, with the radius of the fiber. Using Equations (5) and (6) we have obtained the rate of change of the resonant wavelength with the radius of the fiber as

(17) $$\frac{d\lambda }{d{\tilde{r}}_{co}}=\frac{\lambda_{2}\alpha_{p}}{2^{1/3}3\pi {\left(n_{co}{\tilde{r}}_{co}\right)}^{5/3}}\left[\frac{U_{m,p}}{R_{b}}+\Delta k\left(q+\frac{1}{2}\right)\right]$$

Here ${\tilde{r}}_{co}=r_{co}k_{o}$ is the normalized fiber radius. For all fiber radii $d\lambda_{r}/dr>0$, *λ_r_* increases with the increase in the fiber radius. As one can see in Equation (17) and Figure 6 the rate of change of the resonant wavelength with the radius, $d\lambda_{r}/dr$, increases with a decrease in the radius of the fiber, and this rate $d\lambda_{r}/dr\to 0$ as the radius of the fiber increases substantially. For our structures, where Δ*k*<<*U_m,p_*/*R_b_* Equation (17) can be simplified and presented as

(18) $$\frac{d\lambda_{r}}{d{\tilde{r}}_{co}}\approx \frac{2^{1/3}4\pi n_{co}\alpha_{p}}{3{\left(n_{co}{\tilde{r}}_{co}\right)}^{1/3}{\left[\alpha_{p}+2^{1/3}{\left(n_{co}{\tilde{r}}_{co}\right)}^{2/3}\right]}^{2}}$$

As in the case of the refractive index sensor, the sensitivity of TM polarized modes exceeds the sensitivity of TM polarized modes but these values are comparable (Figure 6). Our temperature sensor with a sensitivity of 10 pm/K can provide a temperature detection limit of 1 K if an OSA with a resolution 10 pm is used for the monitoring process. This sensitivity is comparable tothe sensitivities of other WGM sensors [14].

## 4. Conclusions

We have proposed the use of a bottle resonator as a sensor. We have theoretically analyzed the operation of a bottle resonator with an altitude of several nanometers and with a length of several hundreds of micrometers made on the surface on the fiber with a constant radius, within a range of 10μm and 100μm. Such bottle resonators can be made with CO_2_ laser processing or with 248 nm excimer laser beam ablation with sub-angstrom precision [23]. They can be excited with a tapered fiber placed at the top of the resonator perpendicular to the fiber axis. Like FBG sensors the bottle resonator sensors have all the advantages of the fiber geometry and can be used for refractive index and temperature sensing. Contrary to FBG sensors bottle resonator sensors are immune to decay at high temperature. A bottle resonator made on the fiber surface does not cause coupling of the fiber modes propagating in the core of the fiber, that is bottle resonator sensors can be made on the surface of an active fiber device, such as a high power fiber laser or a laser cooled fiber sample, to monitor the temperature distribution along these devices without any perturbation of device performance. The refractive index bottle resonator sensors have advantages over the SPP sensors, as they are free from metal parts, which introduce undesirable loss in the system. Although the refractive index sensitivity of SPR sensors is higher than the sensitivity of bottle resonator sensors, a bottle resonator sensor with a nanoscale altitude made on the top of the fiber can be easily integrated in any fiber scheme.
